# Post-transplant immunotherapy with *WT1*-specific CTLs for high-risk acute myelogenous leukemia: a prospective clinical phase I/II trial

**DOI:** 10.1038/s41409-018-0383-2

**Published:** 2018-11-08

**Authors:** Hee-Je Kim, Hyun-Jung Sohn, Jung-A Hong, Hyun-Joo Lee, Dae-Hee Sohn, Chang-Ae Shin, Hyun-Il Cho, Woo-Sung Min, Tai-Gyu Kim

**Affiliations:** 1 Leukemia Research Institute, Seoul St. Mary’s Hematology Hospital, College of Medicine, The Catholic University of Korea, Seoul, Korea; 2 Catholic Hematopoietic Stem Cell Bank, College of Medicine, The Catholic University of Korea, Seoul, Korea; 3 Department of Microbiology, College of Medicine, The Catholic University of Korea, Seoul, Korea

**Keywords:** Lymphocyte activation, Immunotherapy

Wilms’ tumor antigen 1 (*WT1*) is more abundant in leukemic cells than in normal hematopoietic cells. Quantitative assessment of *WT1* gene transcript abundance by real-time quantitative PCR (RQ-PCR) has been shown to be useful for predicting clinical outcome and prognosis in acute myelogenous leukemia (AML), and for detecting minimal residual disease (MRD) [1–3]. In addition, the expansion of *WT1*-specific CD8^+^ T cells was correlated with graft-versus-leukemia (GVL) effect in 10 subjects with acute lymphoblastic leukemia [4]. Autologous vaccination of AML patients with *WT1* peptide or with full-length *WT1* mRNA-electroporated dendritic cells (DCs) showed immunogenic and anti-leukemic activity, as evidenced by the conversion of partial remission and the induction of molecular remission [5, 6]. Adoptive transfer of *WT1*-specific T cells mediated antileukemic activity and persistence in relapsed or high-risk leukemia patients after hematopoietic stem cell transplantation (HSCT) [7, 8]. In the present prospective clinical phase I/II study with long-term follow-up, we demonstrated that adoptive transfer of *WT1*-specific cytotoxic T cells (*WT1*-CTLs) generated in vitro from donor-derived DCs transduced with an adenoviral vector expressing human *WT1* (Adv-*WT1*) is a feasible therapeutic tool with acceptable safety that can induce an optimistic long-term clinical response accompanied by T-cell responses against *WT1* in adult patients with relapse high-risk AML after allogeneic HSCT.

A total of 13 newly diagnosed adult patients treated for AML between 2007 and 2008 in the Catholic Blood and Marrow Transplantation Center were considered eligible for this study if they had a human leukocyte antigen (HLA)-identical sibling donor. The trial included five male and five female patients, with a median age of 40 years (range, 28–49 years), who were categorized as high-risk AML mainly based on the higher expression levels of *WT1* at initial diagnosis [2, 9] and received an allogeneic sibling donor HSCT followed by anti-leukemic *WT1*-CTLs infusion (Table 1). For in vitro induction of *WT1*-CTLs from healthy donors, monocyte-derived DCs were transduced with an adenoviral vector for *WT1* expression. The proportion of CD8^+^ and CD4^+^ T cells in the generated CTLs was 65.9 ± 15% and 25.9 ± 12% and the frequencies of *WT1*-specific IFN-γ-secreting CD8^+^ and CD4^+^ T cells were 147.3 and 305 per 10^6^ cells, respectively. Beginning on D+35 post-transplantation, 4 × 10^7^ *WT1*-CTLs were infused four consecutive times at 1-week intervals in patients without moderate to severe acute graft-versus-host-disease (GVHD). Every 1–3 months after the CTLs infusion, and for at least 1 year post transplantation, we serially monitored the clinical status, and the peripheral blood lymphocyte subpopulations using flow cytometry, the in vitro activity of interferon-gamma (IFN-γ) by enzyme-linked immunospot (ELISPOT) assay, and *WT1* expression levels by RQ-PCR.

**Table 1:** Characteristics of enrolled patients

	UPN1	UPN2	UPN3	UPN4	UPN5	UPN6	UPN7	UPN8	UPN9	UPN10
Diagnostic subtype	Hypoplastic	MLD	M7	M1	M2	MLD	M2	M0	M1	MLD
Age: D/R	54/46	37/41	45/43	36/39	26/28	54/49	39/38	31/37	35/30	41/31
Sex: D/R	M/F	M/F	M/F	M/M	M/M	F/M	F/M	F/F	F/F	M/M
Pre-HSCT status	CR1	CR1	CR1	CR1	CR1	CR1i	Untreated Relapse after CR2i	CR2i, CR1 after third induction	Primary refractory	CR1
Cytogenetics	47 XX, +8	46 XX, 1qh+	46 XX,del(1q), −3,−5,+8,+11,−13,−17, 19,3 ~ 3mar	46 XY	46 XY,del(7)	46 XY	46 XY, *t*(8;21)	46 XX, *t*(11:19), der(20), t(20:?)	46 XX, *t*(11:15), add(18)	46 XY,*t*(6;9)
Molecular/IP abnormality	—	CD7+	—	FLT3-ITD+	—	FLT3-ITD+, CD7+	c-kit D816V mutation+	—	—	FLT3-ITD+NPM1wt
WT1 level at Dx	High	High	High	High	High	High	High	High	High	High
AGvHD	No	Yes, grade II	Yes, grade II	Yes, grade I	Yes, grade I	No	Yes, grade II	No	No	No
CGvHD	Yes, extensive	Yes, extensive	Yes, extensive	No	No	No	Yes, extensive	No	No	Yes, limited
TRM	No	Yes	No	No	No	Yes, Sepis	No	No	No	No
Relapse	No	No	No	No	No	No	Yes, chest wall chloroma	Yes, Leukemia cutis	Yes, Leukemia cutis	No
Outcome (as of May 31, 2017)	Alive, 10 y 10 m	Died, 1 y	Alive, 10 y 8 m	Alive, 10 y 7 m	Alive, 10 y 4 m	Died, 10 m	Died, 7 m	Died, 5 m	Died, 4 m	Alive, 9 y 2 m

*MLD* multilineage dysplasia, *D* donor, *R* recipient, *M* male, *F* female, *WBC* white blood cell, *CRi* complete remission with incomplete recovery of CBC, *Dx* diagnosis, *HSCT* hematopoietic stem cell transplantation, *SCs* stem cell source, *IP* immunophenotype, *WT1* Wilms’ tumor gene 1, *AGVHD* acute graft-versus-host disease, *CGVHD* chronic graft-versus-host disease, *TRM* transplant-related mortality, *PBSC* peripheral blood stem cell, *BM* bone marrow, *G-BM* G-CSF-primed bone marrow, *NPM1wt* wild-type nucleophosmin1 gene

All patients were successfully engrafted; however, three of them (UPN 7, 8, and 9) died due to relapse after transplant. One of the two patients with treatment-related mortality (TRM) (UPN 2) died due to septic pneumonia and cytomegalovirus (CMV) disease in the gut combined with extensive-type GVHD at 1 year, and the other patient (UPN 6) died due to rapidly progressing gram-negative sepsis and a disseminated herpes simplex viral infection at 10 months after transplant. However, these two patients had no evidence of relapse. The five other patients are alive, at a median follow-up of 127 months (range, 102–130 years), and the 8-year event-free survival (EFS) rate was 50%. In our previous studies, the long-term survival rates for high-risk patients who received allogeneic stem cell transplantation in CR1 without any adoptive immunotherapy is less likely around 30% [9, 10]. Among patients with complete remission 1 (CR1) pre-HSCT status, the EFS rate was 71.4%. These findings suggest that *WT1*-CTLs administration could induce prolonged remission only in patients without MRD, but not prevent the rapid proliferation of leukemic stem/progenitor cells even after transient hematological CR conditions established by myeloablative conditioning.

Despite the beneficial potential of *WT1*-CTLs therapy following allogeneic HSCT, it is necessary to consider the possibility of inducing severe chronic GVHD related to CTLs infusion. In the study, 4 out of 10 (40%) patients developed extensive type of chronic GVHD. As depicted in Table 1, UPN1 showed de novo type of chronic GVHD with skin and liver involvement and a typical manifestation of sicca 4 months after HSCT. UPN2 also showed a persistent type of chronic GVHD starting from grade I acute GVHD just early after the 3rd infusion of *WT1*-CTLs. UPN3 and UPN7 showed a multi-organ pattern of grade II acute GVHD involving the skin and gut after the final infusion of *WT1*-CTLs, and then progressed to extensive type of chronic GVHD until 6 months, 7 months after transplantation, respectively. Although, the precise causal relationship in association with the infused *WT1*-CTLs was not clear enough, patients having extensive type of chronic GVHD not in relapse were successfully manageable without any long-term sequelae, as shown in Table 1. Further revelation to clarify the direct effect of infused *WT1*-CTLs on chronic GVHD is quite anticipated in the future study.

The results from the long-term monitoring of the five living patients showed individual differences in the frequencies of *WT1*-specific CD8^+^ and CD4^+^ T cells and *WT1* expression levels in peripheral blood mononuclear cells (Supplementary Figure 1). In UPN 1, there were predominant strong-specific T-cell responses with mostly CD4^+^ T cells following three peaks in *WT1* expression. UPN 3 and UPN 4 showed mainly CD4^+^ T-cell responses around a single peak of *WT1* expression. Among the patients with relapse, UPN 8 showed an increased, sustained, strong T-cell response as *WT1* expression increased. Our data suggest that *WT1*-specific T-cell activity increases in response to an increase in the amount of *WT1* antigen expressed by the leukemic cells in the patients, and that CD4^+^ T cells are as important as CD8^+^ T cells in inhibiting leukemia. Also, we measured the lymphocyte subpopulations in the patients’ blood for 140 weeks after the first *WT1*-CTLs infusion using multiparameter flow cytometry (MFC) (Supplement Figure 2). Various clinical features were observed in each patient and the clinical relevance of the prognosis was not observed. Supplementary Figure 1 has shown that *WT1*-CTLs is already present in some AML patients and dose not clearly demonstrate the persistence and efficacy of infused cells. But our previous pilot trial, we observed that frequencies of CD4^+^ T cells and CD8^+^ T cells increased progressively during serial infusion of *WT1*-CTLs and the pattern persisted until 9 months together with specific T-cell responses maintained against *WT1* for more 2 years infusion [8]. In other previous study has shown that gene marking studies have been performed to identify cytotoxic T-cells infused after HSCT [11]. In this study, it was difficult to determine whether the engraftment and proliferation of infused cells. Therefore, further studies will be required to confirm the efficacy of infused donor cells by short tandem repeat (STR) test.

Taken together, despite the small number of patients enrolled in the study, this exploratory trial of *WT1*-CTLs after allogeneic HSCT for high-risk AML suggests the possibility of using this therapeutic strategy in combination with elective allogeneic or even autologous HSCT with *WT1*-CTL infusion. Generation of multi-antigen-specific T cells to enhance the GVL effect and prevent infection after allogeneic HSCT may be promising treatment in the near future, as well as the development of immunotherapy using T-cell receptor (TCR)-engineered T cells [12, 13].

## Electronic supplementary material

Supplementary Information
