# Supplementary material for: Post-transplant immunotherapy with WT1-specific CTLs for high-risk acute myelogenous leukemia: a prospective clinical phase I/II trial
Source: Bone Marrow Transplant. 2018 Nov 8;54(6):903–6. doi: 10.1038/s41409-018-0383-2 (PMC6760543; doi:10.1038/s41409-018-0383-2)

**Supplementary Information**

**Supplementary figure 1. Individual immune responses after *WT1*-CTLs therapy.** Black bars indicate the numbers of IFN-γ secreting cells per 10^6^ CD8^+^ T cells and white bars indicate the numbers of IFN-γ secreting cells per 10^6^ CD4^+^ T cells over time. Blue indicates the plasma *WT1* transcript copy numbers after *WT1*-CTLs infusions. The arrows (↑) indicate the *WT1*-CTLs infusions, and the stars (*) indicate when the patient died.

**
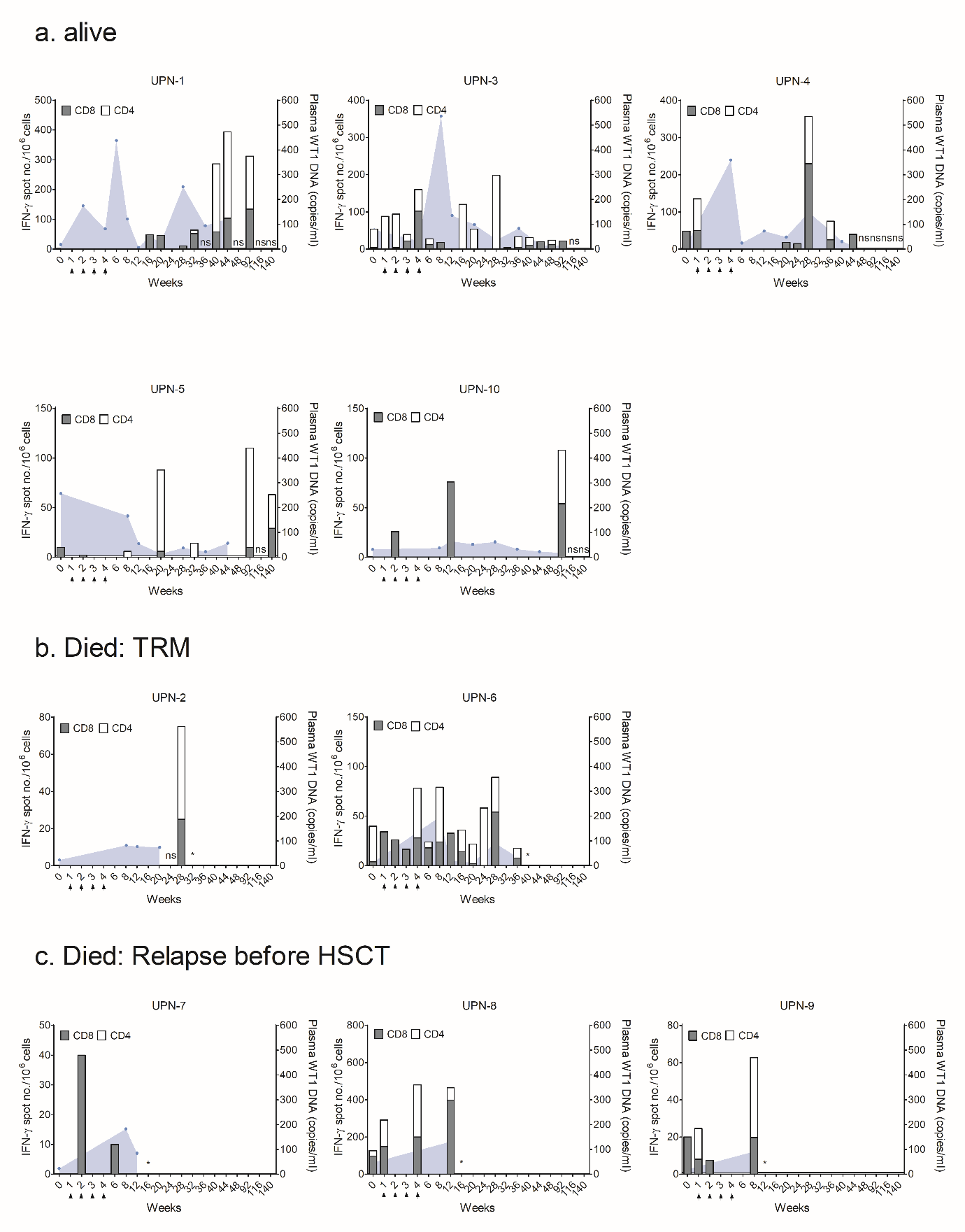
**

**Supplementary figure 2. Immunophenotype analysis of PBMCs in patients after *WT1*-CTLs therapy.** (a) Alive, (b) Dead: TRM, and (c) Dead: relapse before HSCT. The arrows (↑) indicate the *WT1*-CTLs infusions, and the stars (*) indicate when the patient died.


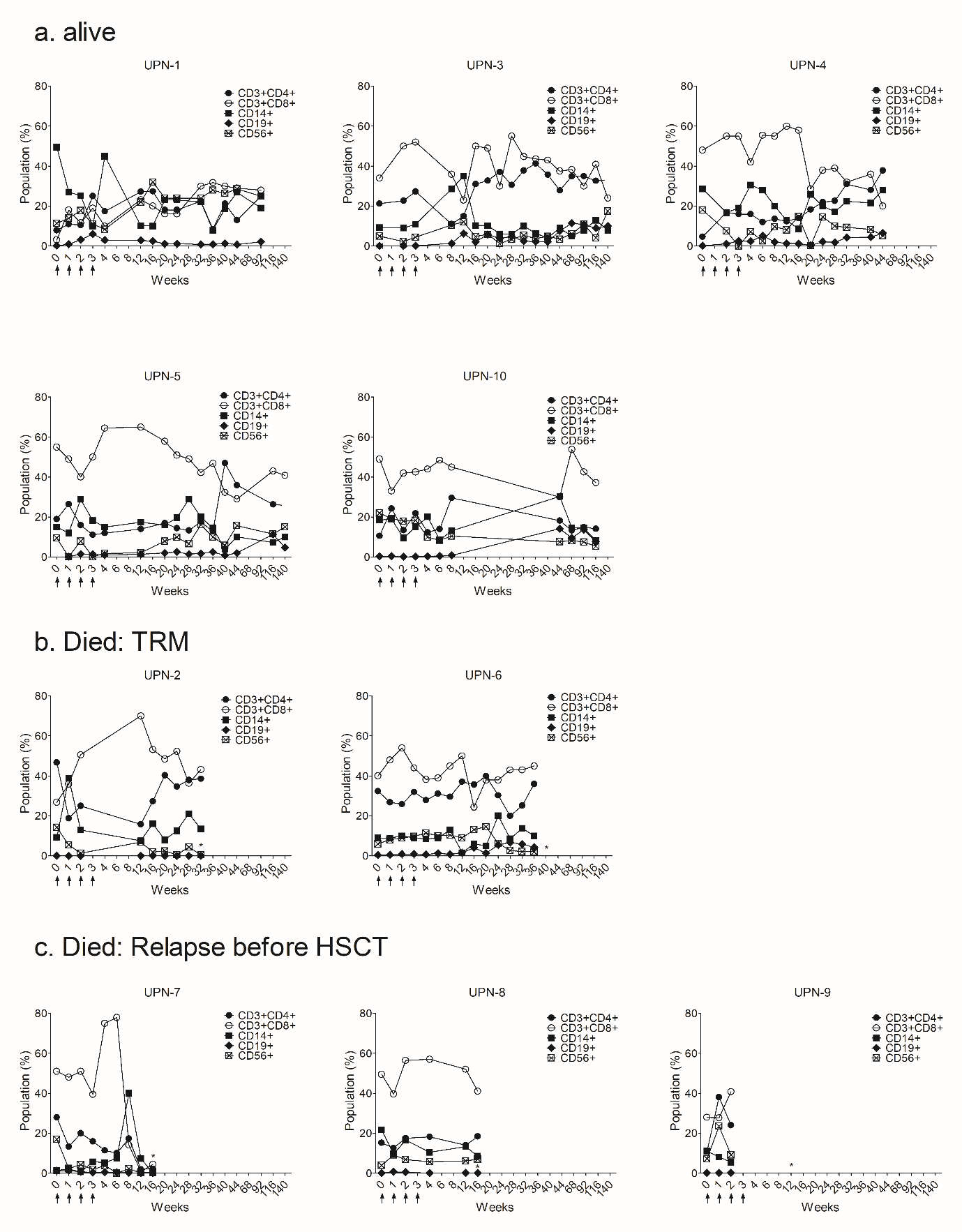

Supplement: Supplementary file 1 — Supplementary Information [file 41409_2018_383_MOESM1_ESM.docx]
